# Effective Program Management: A Cornerstone of Malaria Elimination

**DOI:** 10.4269/ajtmh.14-0255

**Published:** 2015-07-08

**Authors:** Jonathan Gosling, Peter Case, Jim Tulloch, Daniel Chandramohan, Jennifer Wegbreit, Gretchen Newby, Cara Smith Gueye, Kadiatou Koita, Roly Gosling

**Affiliations:** Business School, University of Exeter, Exeter, United Kingdom; Bristol Business School, University of the West of England, Bristol, United Kingdom; Malaria Elimination Initiative, Global Health Group, University of California, San Francisco, California; London School of Hygiene and Tropical Medicine, London, United Kingdom

## Abstract

Effective program management is essential for successful elimination of malaria. In this perspective article, evidence surrounding malaria program management is reviewed by management science and malaria experts through a literature search of published and unpublished gray documents and key informant interviews. Program management in a malaria elimination setting differs from that in a malaria control setting in a number of ways, although knowledge and understanding of these distinctions are lacking. Several core features of successful health program management are critical to achieve elimination, including effective leadership and supervision at all levels, sustained political and financial commitment, reliable supply and control of physical resources, effective management of data and information, appropriate incentives, and consistent accountability. Adding to the complexity, the requirements of an elimination program may conflict with those of a control regimen. Thus, an additional challenge is successfully managing program transitions along the continuum from control to elimination to prevention of reintroduction. This article identifies potential solutions to these challenges by exploring managerial approaches that are flexible, relevant, and sustainable in various cultural and health system contexts.

## Introduction

Effective program management is essential to ensure the elimination and eventual eradication of malaria.^1^ Malaria elimination, defined as the interruption of local transmission in a specific geographical area,^2^ is a long-term, focused, and technical process that requires effective management and communication at all levels. There are several core features of successful health program management, all of which are critical to achieve elimination. In general, elimination is facilitated by robust health systems, determined leadership, appropriate incentivization, an effective and real-time surveillance system, and regional collaborations. As with all aspects of health program management, elimination is hampered by inflexible health systems, a lack of sustained political and financial commitment, ill-equipped managers, unmotivated and untrained staff, and external donor constraints.

Program management in a malaria elimination setting differs in a number of ways from program management in a malaria control setting, and there is currently a lack of research and thorough understanding of these distinctions. In several respects, the requirements of an elimination program conflict with those of a control regimen^3^; thus, an additional challenge is successfully managing smooth transitions along the continuum from control to elimination and beyond to prevention of reintroduction.

On the basis of management science and malaria elimination research, this article brings a new perspective to what the medical sciences sees as an intractable problem: the problem of poor delivery of efficacious interventions that hampers public health goals such as disease elimination and eradication. This perspective offers specific recommendations to address the management challenges that arise along the continuum from control to elimination to prevention of reintroduction. An in-depth account of our analysis and findings can be obtained in the full report, *Program Management Issues in Implementation of Elimination Strategies,* the Global Health Group, University of California, San Francisco Global Health Sciences (http://globalhealthsciences.ucsf.edu/sites/default/files/content/ghg/mei-program-management-issues.pdf).^4^ Here, we present a summary of the most salient findings, informed by published and gray literature on malaria elimination, other disease eradication, and general health program management topics. In addition, we conducted 15 key informant interviews with malaria field experts and members of malaria control and elimination programs, as well as experts in the eradication of diseases other than malaria. Key informants had a range of backgrounds and specialties, and included malaria program managers, academic researchers, and technical experts and advisors at various global public health agencies.

## Findings

### Shifting from control to elimination.

Although the aim of malaria control is to reduce morbidity and mortality in the general population through improved access to prevention, diagnosis, and treatment, elimination requires a case-by-case focus, finding and treating symptomatic and asymptomatic infections alike, and taking action in specific foci to immediately prevent onward transmission.^3^,^5^ Rigorous case investigation and reactive case detection activities that do not generally occur in control settings are necessary in elimination settings to track secondary cases that arise in these foci. Similarly, in control settings, an universal coverage of vector control interventions is often a goal, whereas elimination narrows the focus to high-risk groups based on demographics, or “hotpops,”^6^ and malaria high-risk micro-foci, or hotspots,^7^ based on geography where cases and ongoing transmission are concentrated. Prevention of reintroduction depends upon a program's ability to continue targeted vector control measures and active case detection among high-risk groups, and maintain the ability of the health system to recognize malaria cases and rapidly contain outbreaks.

A malaria program's transition from control to elimination is often publicly heralded as a formal reorientation with a commitment to a time-specific goal. In reality, however, the transition is more of a continuum; as transmission declines in some geographical areas, elimination strategies can be implemented, and where malaria transmission remains high, control measures continue to be pushed. Thus, many countries exist in both control and elimination phases concurrently.

Throughout the transition, the degree to which interventions are integrated in the local health system may vary. Generally, in the early stages of elimination, program investments are targeted toward strengthening capacity to deliver vertically controlled services. However, the long-term resilience of malaria elimination programs depends on the integration of this capacity into local health systems. As case incidence declines, governments and external sponsors tend to taper funding and the malaria program relies solely on integrated services within the general health system.^8^ The timing and degree of program integration must be context-specific, and a balanced approach ameliorates the risks associated with either an entirely vertically controlled program, subject to changing domestic funding priorities, or a fully integrated program, which may sideline malaria elimination activities in favor of more pressing health challenges. Ultimately, managing the interface between integrated health systems and vertically controlled programs requires considerable sensitivity. Success is dependent upon well-maintained relationships between those guiding the elimination effort and stakeholders at all levels, and a long-term commitment to securing appropriate financing.

### Enabling factors for elimination.

The following enabling factors contribute to a successful malaria elimination program.

#### A robust local health system.

A strong health system provides both a downward flow of data, requisite policies, personnel, and materials, and an upward flow of data and management feedback on what is working and what is not. Elimination programs may enhance this system in three ways: 1) enable system-wide access to data on prevention, diagnostic test results, treatments, and responses; 2) provide specialist teams and supplies to intervene in every individual case; and 3) sustain long-term attention to malaria even when cases fall to zero. These enhancements require subnational administration units and a focused, tailored response package to be maintained over a period of 6–10 years.^3^

#### Leadership.

At the provincial, district, and village levels, leadership takes the form of motivated and inventive people able to solve practical problems of supply, funding, and personnel; adapt to unforeseen events; mediate between the sometimes conflicting priorities of the vertical and integrated systems; and maintain focus on the key tasks of surveillance and response. Effective leadership also empowers lower level staff to make decisions and initiate action without constant directives and input from program managers, thus encouraging local ownership and minimizing response delays. At the national level, long-term, sustained leadership ensures institutional memory and continuity, and maintains focus on and political power for malaria elimination well beyond its popular urgency. At this level, leadership must have direct ties to other ministries (health, finance, development, agriculture, etc.) and ideally have strong support from the Head of State.

#### Incentives.

Successful elimination can only occur when personnel at all levels are effectively engaged. Once a decision has been made to start planning for elimination, incentives can be aligned with more targeted interventions, rewarding swift attention to individual cases. Incentives can be independent of normal arrangements for pay and do not need to be monetary, but must enhance the value of malaria elimination work to the individuals involved, and be sustainable for 6–10 years.^9^,^10^

#### Regional collaborations.

Preventing and responding quickly to imported malaria is critical both during and after the elimination phase.^5^,^11^ This requires binational or multinational collaboration to share information on population movement and the occurrence of malaria in border regions, and to coordinate interventions to prevent reintroduction of malaria. At an operational level, such collaboration requires personnel who are authorized and willing to share information across borders and respond jointly with prevention measures that can be adapted within different national health systems. Ministerial-level leaders must support this collaboration.

#### A framework of organizational learning and evaluation.

Elimination activities should be guided by a formalized organizational learning structure. This includes developing metrics to gauge success and failure, but goes further in convening stakeholders where outcomes can be interpreted and lessons identified to improve practices. Managers must do more than administer established protocols; with appropriate support they must reflect on their practices, assess the reasons for success or failure, and devise contextually appropriate solutions. In addition, annual micro-planning of elimination activities and frequent monitoring of targets and responses are essential.

### Roadblocks to elimination.

Although management challenges differ from country to country and region to region, it is possible to identify some common problems.

#### Systemic roadblocks.

Systemic roadblocks such as institutional conservatism and a lack of political commitment will hamper elimination efforts. Introducing new approaches for elimination requires creative thinking and an ability to change existing structures and practices from the top governmental levels downward.

#### Nonspecialist managers and administrators.

The appointment of personnel who have little or no technical understanding of malaria to positions of authority over the malaria elimination effort is a common problem. Elimination is a highly technical process, and managers without the appropriate skills may make ill-informed decisions, in turn decreasing the respect and motivation of technical staff responsible for operational implementation. Yet, both technical and nontechnical leaders will have their strengths and weaknesses. Nontechnical leaders must be willing to listen to their technical staff when scientific input is needed; leaders with technical expertise may require more dedicated program management support through training or human resources. Whatever the leader's background, he/she must be able to articulate and represent program goals that transcend the particular interests of either managerial or technical stakeholders. All staff must feel that their leader is one of them and speaks for them.

#### External donor constraints.

Of the 34 malaria eliminating countries (as of 2014), 20 received external funding from the Global Fund to Fight acquired immune deficiency syndrome, Tuberculosis and Malaria (GFATM) between 2005 and 2012.^12^ Compared with the level of funding received by malaria control countries, these grants were often small. These may, in time, prove to have been catalytic in initiating a shift in domestic commitment to elimination. However, this external funding is often tapered at the most critical moment as countries approach elimination and are under increasing internal pressure to reallocate the national malaria budget to higher priority public health programs.

In addition, a reliance on external funds may introduce human resource challenges. The problems associated with foreign-funded staff appointments are common to many aid programs, and include using different pay scales and being time-limited. These situations can ultimately result in a drain of skills and motivation as well as reduced program sustainability when funding ends. In collaboration with other stakeholders, program managers need to strive to overcome these constraints and ensure that external funds promote the long-term goals of the malaria elimination strategy.

### Application of managerial practices.

Applying new technical and behavioral modes of operation can be difficult, and the challenges will differ in each local setting and will likely change over time. Therefore, managerial approaches such as “action learning” and “organizational learning,” approaches that stress continuous adaptation to new information, should be applied.^13^,^14^ For example, health workers who are accustomed to malaria control strategies may not be prepared for the intensive targeting and urgent responses required in the early phases of elimination. Similarly, district-level managers who have succeeded in directing rapid response teams may struggle to sustain resources and personnel for later elimination phases. Because management requires a high degree of cooperation from “the managed,” behavioral change is far more effective when introduced consensually and collectively, drawing on the ideas and skills of all people involved and encouraging two-way engagement. At minimum, using a participatory methodology will ensure better local adaptation and buy-in.

## Discussion

This article draws upon experiences from malaria elimination and general public health programs and management sciences to reflect on the important and under-researched issue of improving malaria intervention delivery through effective program management. Systems to do this exist in other health and development sectors as well as the private sector, yet malaria specialists, who tend to work within traditional confines of vector and parasite control, are often unaware of these systems. Below are key recommendations that arise from this review that support the improved effectiveness of program management along the continuum from malaria control to elimination.

### Assessment of management challenges.

Any changes in practice need to be based on an assessment of existing management skills and capacities in countries that are considering or are ready to transition from control to elimination. This assessment would identify strengths and weaknesses, establish the degree of readiness for the control-elimination transition, and identify where management practices and/or capacity could be improved. Such an assessment should be complemented by research and development into malaria elimination management. Because there is currently a dearth of research focusing specifically on malaria program management issues, particularly at the operational level, investment in practice-oriented research that is situation-specific and actively engages managers on the front line is essential.

### Building management capacity.

We recommend three interrelated actions to enhance capacity for malaria elimination. First, investment in leadership development programs to support the transition from control to elimination at all levels of a health system is critical. The type of leadership training will vary according to the role of personnel and the level at which they operate. Second, there is a need for management development at the operational level, particularly team building and team management skills and techniques. Senior technicians should be given management training in finances, information, logistics, activity planning, and other administrative skills to support systemic improvements to managerial practice. At provincial and district levels, program leaders should be identified and supported with mentoring and training which would assist them in adapting to the changing priorities of the elimination phase. The emphasis in the longer term will shift from gathering and interpreting information and directing rapid, comprehensive responses to maintaining accurate diagnostics and surveillance that can detect increasingly rare cases. Third, organizational learning and capacity building workshops would significantly impact elimination efforts. Continuous assessment of practices and outcomes is an important part of organizational learning, fostering a problem-solving and pro-active approach. Workshops and/or exchange visits for key operators within a country or region would facilitate such networked learning.

### Maintaining performance.

We recommend three human resource strategies to enhance and maintain performance. First, improve staff motivation by implementing appropriate incentive systems. To attain elimination, maintaining an organizational culture in which people want to do the work is essential. Success is more likely if staff members internalize program goals and are motivated to achieve them. Developing non-monetary incentives in addition to paying a living wage should contribute to culturally and contextually appropriate incentives for health workers. Second, improve accountability systems through adoption of locally acceptable performance management procedures. A participatory approach to designing and implementing performance management should be used to ensure long-term local buy-in. Third, address gaps in information management and use of data. Improving data quality and reaching agreement on the minimum essential data needs for elimination should be a priority. If a malaria information system is not equipped to investigate at the household level, achieving elimination is not realistic. Where such systems are in place, the lowest levels of elimination programs should be empowered to act autonomously in response to information as soon as it is available.

### Piloting management reforms for malaria elimination.

Reorientation from control to elimination requires a particular mix of vertically directed and integrated activities that are implemented within a specific management framework suitable for the elimination endgame. The key features of such a framework are: political and operational leadership, centralized quality standards and decentralized quality control processes, systematic learning, and adaptation to local circumstances, flexible human resource practices that sustain continuity of effort. The rigor required for implementation in this framework may best be achieved by contracting out some services to non-Ministry of Health entities. Although there has been some research on the effectiveness of contracting out health services,^15^–^17^ this has not focused on the vertical components of disease control programs, and such research is needed.

### Limitations.

In this perspective article, we have examined an important topic on which scientific evidence is problematic because of a basic shortage of reliable empirical studies on malaria program management. The findings and recommendations draw on both published and unpublished literature and qualitative data gathered from key informant interviews with malaria elimination experts. Formal systematic review methods and qualitative analysis based on the key informant interviews were not carried out. The findings presented are the opinions of the authors based on the synthesis of decades' worth of key informant expertise, review of the available evidence supported by management research in other fields, and should serve to advance the knowledge base and stimulate research on the neglected field of malaria program management.

## Conclusion

Approaching malaria elimination with “business as usual” attitudes and expectations is untenable. Malaria elimination is a long-term, focused, and technical process that requires effective management and communication at all levels. The analysis and recommendations we present in this article provide a way to improve effectiveness of elimination management through building and enhancing existing strengths while offering realistic suggestions for changes in practice and further research to tackle the remaining challenges.
